# Divergent CD27 expression marks the Treg induction trajectory

**DOI:** 10.3389/fimmu.2026.1756275

**Published:** 2026-03-09

**Authors:** Chelsea Gootjes, Maurits G. Staal, Diahann T. S. L. Jansen, Antoinette M. Joosten, Jaap Jan Zwaginga, Bart O. Roep, Tatjana Nikolic

**Affiliations:** Department of Internal Medicine, Section Immunomodulation and Regenerative Cell Therapy, Leiden University Medical Center, Leiden, Netherlands

**Keywords:** CD27, immunotherapy, induced Tregs, tolerance induction, Treg development

## Abstract

**Introduction:**

The induction of antigen-specific Tregs is explored as strategy to restore immune tolerance and halt progression of autoimmune diseases. However, the phenotypic changes in development of induced antigen specific Tregs *in vivo* have not been defined extensively. CD27 expression marks superior suppressive naturally occurring Tregs (nTregs) while in cancer, this showed to be a prognostic marker for tumor progression. Tumors indeed can promote the immunosuppressive effects of the CD27-CD70 co-stimulatory axis, and CD27 has been a target for immune checkpoint blockade in cancer. In this study, we explored changes in CD27 expression along with a panel of markers associated with immune regulation.

**Methods:**

For this, we induced Tregs *in vitro* from naive CD4 T cells by tolerogenic dendritic cells (tolDCs) and compared their phenotypes to effector T cells induced in parallel cultures by pro-inflammatory mDCs in time following priming.

**Results:**

Clustering analysis revealed three clustering groups distinguishing induced Treg cultures from effector T cells, all marked by high CD27 expression, of which two clusters had a memory-like phenotype and expressed regulatory markers TIGIT, PD-1 and CD38. The kinetics of CD27 expression showed that naive T cells increase CD27 expression during their differentiation into memory-like Tregs, whereas CD27 is lost during differentiation into proinflammatory effector T cells. Furthermore, the presence of CD27 and TIGIT expressing memory-like Tregs positively correlated with the inhibition capacity of the induced Treg lines *in vitro*. Increased ratios of these Tregs over effector T cells *in vivo* following vaccination of T1D patients with tolerogenic DCs pulsed with islet autoantigen correlated with increased islet-specific immune regulation ex vivo.

**Discussion:**

Our results define a population of induced Tregs *in vitro* and *in vivo* that is marked by elevated CD27 expression. Hence, CD27 expression may be useful to monitor therapeutic efficacy of Treg induction *in vivo* in clinical trials.

## Introduction

In autoimmune diseases the impaired central or peripheral tolerance toward self-antigens unleashes unnecessary attacks by the immune system (1–3). Peripheral tolerance critically relies on regulatory T cells (Tregs), which can develop in the thymus (nTregs) or be induced in the periphery (iTregs) (4–6) and suppress the immune system selectively by a range of different mechanisms (7). CD25, TIGIT, PD-1 and absence of CD127 commonly mark nTregs, while CD45RA expression discriminates naive from memory Tregs. More detailed characterization of nTregs based on their surface phenotype revealed 22 different subsets (8). In contrast, the detection of adaptive iTregs *in vivo* is not yet specified and phenotypic changes following priming of antigen-specific iTregs are poorly defined. An interesting marker in this regard is CD27, a member of the tumor necrosis factor receptor superfamily that is expressed on naïve T cells and increased on memory T cells upon activation (9). CD27 expression was also reported on expanded human nTregs and correlated with superior regulatory activity (10, 11). CD27 received emerging interest as a target for immune checkpoint blockade [reviewed in (12, 13)]. Tumors employ the CD27-CD70 axis to modulate the immune system; dysregulation of this axis is associated with tumor progression and immunosuppression (14, 15). CD70 is expressed by malignant solid tumors and by hematological malignancies in combination with CD27 (14–18). CD27 expression on T cells in the tumor microenvironment of multiple myeloma patients is a negative prognostic marker for patient survival (19). Also, high levels of soluble CD27 have been found in sera of patients with malignancies.

While CD27 was described as an immunoregulatory molecule on nTregs and in the tumor microenvironment, little is known about CD27 expression upon induction of iTregs. In this study, we induced autoantigen-specific Tregs from naïve CD4 T cells by tolerogenic dendritic cells (tolDC) *in vitro* and monitored CD27 expression during Treg development in comparison with effector T cells (Teff) induced by pro-inflammatory mDCs.

## Methods

### Generation of monocyte-derived dendritic cells

Buffy coats of 17 healthy human donors were purchased from the blood bank Sanquin following informed consent. Peripheral blood mononuclear cells (PBMCs) were isolated from these buffy coats with Ficoll density gradient centrifugation. Monocytes were isolated from these PBMCs to generate tolDC and proinflammatory mDCs as described previously (20). In short, monocytes were isolated with a positive selection using CD14 beads (Miltenyi) according to the manufacturer’s protocol and cultured in RPMII medium (Gibco) supplemented with 8% FCS (Hyclone), IL-4 (500 IU/ml, Miltenyi) and GM-CSF (800 IU/ml, Miltenyi) for 6 days. Medium and cytokines were refreshed on day 3 of culture. To generate tolDCs, vitamin D3 (10–^8^ M, Carbogen Amcis, day 0 and 3) and dexamethasone (10–^6^ M, Centrafarm, day 3) were added to the medium. Immature dendritic cells were matured with a cytokine mix containing GM-CSF (800 IU/ml), IL-6 (500 IU/ml), IL-1beta (1600 IU/ml), TNF-alpha (335 IU/ml) all from Miltenyi and PGE2 (2 ug/ml, Pfizer) for two days after which they were pulsed with beta-cell derived antigens (proinsulin peptide C19-A3 or the insulin neoantigen INS-DRiP protein) and used for T cell co-cultures and suppression assays.

### Generation of regulatory and effector T cells

Tregs and Teff cells were induced from each independent donor as described previously (21). In short, naïve CD4 T cells were isolated from the unlabeled cells remaining after isolation of monocytes, by negative selection using the untouched naïve CD4+ T cell Isolation Kit II (Miltenyi) according to the manufacturer’s protocol. Isolated cells were cocultured with autologous antigen-pulsed mature tolDCs or mDCs in a T:DC ratio 10:1, in IMDM (Gibco) supplemented with 10% human serum (HS, Sanquin) to induce antigen-specific T cell lines. After 5 days of coculture, T cells were recovered and rested in the presence of IL-7 (10 ng/ml, Peprotech) and IL-15 (5 ng/ml, R&D Systems) for 2 days. After the 2-day resting period, T cells were harvested again and restimulated with autologous tolDCs or mDCs for 5 days followed by a 2-day resting period again, yielding a regulatory T cell line (iTregs, primed by tolDC) and a matching effector T cell line (iTeff, primed by mDC) per donor. C19-A3 specific iTreg and iTeff lines were induced from all 17 donors. From one donor, additional iTreg and iTeff lines were induced against the islet neoantigen INS-DRiP to analyze the generation using a different target antigen.

### Phenotype analysis of T cell lines

Multiple times during the induction of iTregs and iTeff lines, phenotype analysis was performed. On day 0, 5, 7, 12 and 14 samples were taken for phenotype analysis with spectral flow cytometry. T cells were stained with a surface panel (**Supplementary Table 1**) for 20 minutes at room temperature in the dark. After this incubation cells were washed in PBS and stained with Live/Dead Blue for 15 minutes at room temperature in the dark. Cells were then washed again in FACS buffer (PBS containing 0.5% Albumin (Alburex20) and 0.01% Sodium azide [LUMC Pharmacy)] and taken up in FACS buffer for acquisition on a Cytek 5 laser Aurora Spectral Flow Cytometer (Cytek Biosciences) using the SpectroFlo Software v2.2.0.2. FCS files were analyzed in OMIQ (Dotmatics). The same cryopreserved peripheral blood lymphocytes (PBL) sample was thawed and taken along with every staining to adjusted for differences due to staining on different days (OMIQ function: CytoNorm followed by PeacoQC). In the culture samples, first CD4 T cells were gated by excluding doublets, dead cells, CD14+, CD56+ and CD19+ cells (**Supplementary Figure 1**). Then cells that were CD3+ and CD4+ were used for further analysis. An optsne analysis was performed on equal numbers of CD4 T cells stimulated by either tolDC or mDC on day 14 samples. A group of clusters that were specific to a line were gated manually and a heatmap was made with the clustered heatmap function to show the expression of different markers within these clusters. Furthermore, a UMAP was made with samples of all timepoints and naïve CD4 T cells on day 0 as starting population to perform a branching and trajectory analysis using the Wishbone function (22).

In our pilot experiments, a separate intracellular staining of FOXP3 was performed in iTreg and iTeff lines of nine independent donor using sample taken on day 14 of culture. T cells were fixed and permeabilized with the PerFix-nc Kit (Beckman Coulter) according to the manufacturer instructions. After fixation and permeabilization, cells were stained with a intracellular staining panel (**Supplementary Table 2**) for 30 minutes at room temperature in the dark. Cells were then washed in R3 buffer (included in the kit) and taken up in R3 buffer for acquisition on a Cytek 3 laser Aurora Spectral Flow Cytometer (Cytek Biosciences) using the SpectroFlo Software v2.2.0.2. FCS files were analyzed with FlowJo (version 10.9.0). Technical limitations and permeabilization required for FOXP3 staining prevented us to directly align FOXP3 expression with surface markers.

### Analysis of CD70 expression by DCs

Transcriptomes of tolDCs and mDCs generated in our previous study (23), were used to determine CD70 RNA levels (expressed as Reads Per Kilobase per Million mapped reads (RPKM)). To analyze the surface protein CD70 expression on dendritic cells, mature tolDCs and mDCs were incubated with a CD70-BUV737 antibody (clone Ki-24, BD Bioscience) or with a Mouse IgG3k- BUV737 (clone J606, BD Bioscince, isotype control) for 30 minutes at 4 °C in a 96 wells plate (Costar). Cells were measured on a Cytek 5 laser Aurora flow cytometer (Cytek Biosciences) using the SpectroFlo Software v2.2.0.2. FCS files were analyzed with FlowJo (version 10.9.0).

### Suppression assay

On day 14 of culture, primed iTreg lines were harvested and without preselection tested for their suppressive capacity. Autologous naïve CD4 T cells were used as a responder (Tresp), labeled with 1µM CFSE (Invitrogen) and cultured for 4 days together with iTregs and mDCs loaded with the Treg-cognate antigen (ratio Tresp: Treg : DC 10:10:1) in IMDM/10%HS in a 96 well round bottom plate (Costar). Prior to the assay, plates were coated for 2 hours with a suboptimal concentration of anti-CD3 (0.3 ug/ml, UCHT1, Biolegend), which does not cause T cell proliferation alone but promotes mDC-induced proliferation of CFSE-labeled naïve Tresp, creating a good window to detect Treg-induced inhibition. Irradiated autologous naïve CD4 T cells were included in the wells without Tregs or Teff cells as a crowding control. CFSE-labeled Tresp cells cultured without mDC were used as unstimulated control. After 4 days cells were harvested and stained with anti-CD4-BV650 (20 min. at RT, clone SK3, BD Bioscience) for analysis on a Cytek 5 laser Aurora Spectral Flow Cytometer (Cytek Biosciences). Division of responder cells was used to calculate an expansion index (EI) as described previously (24). An EI of 1.0 (no division) was considered as no proliferation, while the proliferation (EI) of the Tresp stimulated with mDC in the presence of crowding control was considered as maximal proliferation. Inhibition was calculated as percentage of maximal proliferation (100%).

### *In vivo* detection of CD45RA- CD27+ TIGIT+ Tregs

A T cell population with phenotype comparable to that of Tregs induced by tolDCs *in vitro* was defined in our data set obtained during the safety and feasibility trial (D-Sense), in which autologous tolDCs pulsed with proinsulin peptide (C19-A3) were administered to type 1 diabetes patients (25, 26). For this, FCS files from the CyTOF analysis of PBMCs collected at different timepoints after tolDC treatment (25), were reanalyzed using OMIQ software (Dotmatics). IL-10 was measured using an ELISPOT assay, to quantify the number of cells producing IL-10 in the response to the vaccine peptide as described (25).

### Statistical analysis

Graphs were made and statistical significance was analyzed with GraphPad Prism (version 8.0.2). A Two-way ANOVA was performed to test for significant differences in surface marker expression between iTregs and iTeff. To correct for multiple testing a Sidak test was performed. Paired student T test was used to analyze differences in log-transformed mean fluorescence intensity (MFI) of CD27 between iTregs and iTeff or CD70 between tolDC and mDC lines from the same donor. Correlation between abundance of CD27+TIGIT+ iTregs and their suppressive capacity *in vitro* was analyzed with linear regression analysis. Furthermore, unpaired student T test was used to analyze changes in ratio Treg/Teff *in vivo* comparing patients who demonstrated increased or decreased/unchanged IL-10 production by PBMCs in response to the proinsulin vaccine peptide.

## Results

### Induced memory-like Tregs acquire increased CD27 expression compared to iTeff

**Figure 1:**
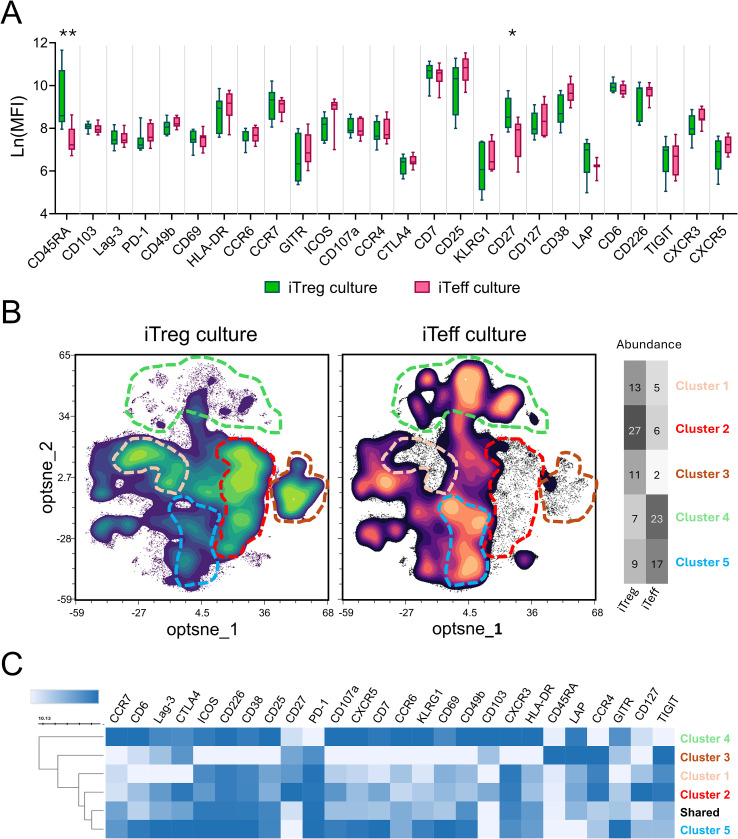
TolDC induced T cell lines (iTreg) show higher CD27 expression, especially in memory-like cells as compared to mDC-induced T cell lines (iTeff). **(A)** Mean fluorescence intensity of surface markers measured in 14 iTreg cultures and 14 corresponding iTeff cultures, on day 14. A Two-way ANOVA was performed to test for significant differences in surface marker expression between iTreg and iTeff cells. A Sidak test was used to correct for multiple testing. Asterisks indicate significance levels: *p<0.03 and **p<0.0001. **(B)** Contour plots of the clustering analysis (optnse) of three iTreg and corresponding iTeff cultures generated in the same experiment. Differential clusters between iTreg and iTeff cultures are indicated with dotted lines: for iTreg (cluster 1-3) orange, red and pink dotted line; for iTeff (cluster 4 and 5) green and blue dotted line. Cluster abundance (%) per culture type is given in the heatmap. **(C)** Heatmap showing the phenotype of differential clusters as gated in B) and the clusters that are equally present in both the lines (Shared). The shades of blue in the heatmap depict per marker the average marker expression ranging from none (white) to high expression (dark blue).

We designed a panel of twenty-six surface molecules including CD27 that discriminate between T cell activation stages or have been associated with either effector or regulatory function to analyze changes in phenotypes of iTreg and iTeff cultures after two weeks from their priming (**Supplementary Table 1**). At the end of the culture period the mean fluorescence intensity (MFI) showed consistently higher expression levels of CD45RA and CD27 in iTreg lines than iTeff lines, whereas most of the other tested markers were similar between iTreg and iTeff cultures (**Figure 1A**). Separate intracellular FOXP3 staining showed no difference in FOXP3 abundance and expression levels between iTreg and iTeff lines at day 14 of culture (**Supplementary Figures 2A, B**). To assess differential marker expression at the single-cell level, an unsupervised clustering including all 26 surface markers was performed (**Figure 1B**). This revealed three cluster nodes uniquely present in iTreg lines and two clusters more abundantly present in iTeff than in iTreg cultures (**Figure 1B**). These five differential clusters were gated and analyzed separately to further explore their phenotypes (**Figure 1C**; **Supplementary Figure 3**). The three clusters distinctive for iTreg cultures all expressed CD27; two contained memory-like T cells (CD45RA- CCR7-), and expressed the regulatory markers TIGIT, PD-1 and CTLA4 in combination with high CD27, while the other one co-expressed CD45RA (further referred to as naïve-like T cell population (**Figure 1C**; **Supplementary Figure 3**). The presence of distinctive CD27+ clusters within iTreg cell lines induced by tolDCs was confirmed in a cluster analysis on three independently generated cultures using different donors (**Supplementary Figure 4**).

### Two branches of differentiating iTregs and iTeffs show different phenotypes

**Figure 2:**
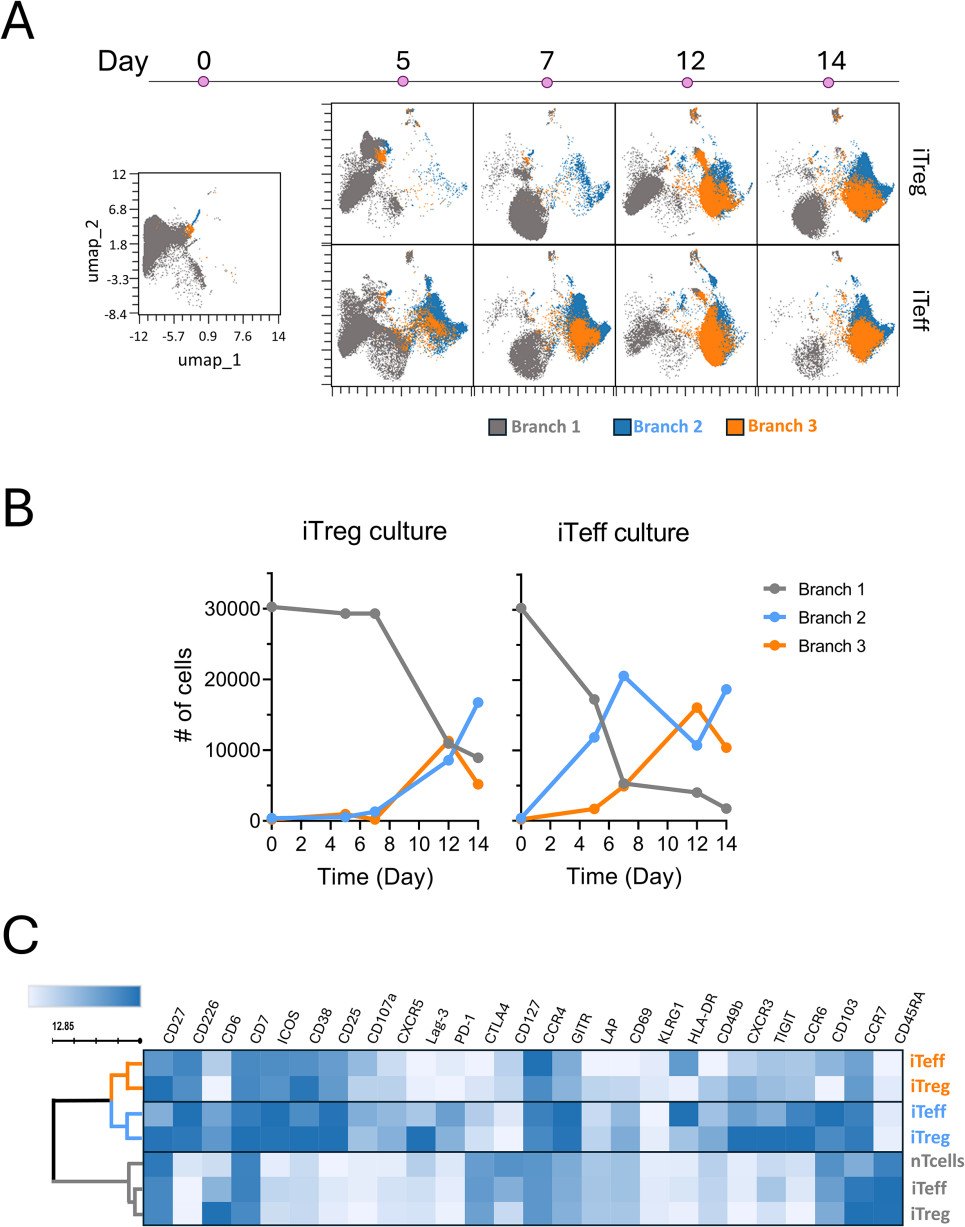
iTregs and iTeff lines follow the same branching, but the phenotype of cells within each branch differs between iTregs and iTeffs. Wishbone branching analysis based on the phenotype of iTreg and iTeff cells from a representative donor, performed using the samples taken from the cultures at different timepoints during the induction. Distribution of cells within each branch per timepoint for iTregs and iTeff cells presented as **(A)** UMAP plots or **(B)** the cell abundance of the three branches. **(C)** Heatmap showing the average marker expression for iTreg or iTeff cultures within each branch (all timepoints together; branch 1=gray, branch 2= blue and branch 3= orange). The shades of blue in the heatmap depict per marker the average marker expression ranging from none (white) to high expression (dark blue).

To further explore changes in T cell phenotypes during tolDC- or mDC-induced differentiation, we applied the Wishbone algorithm that identifies bifurcating developmental trajectories from single-cell data. We used samples taken at multiple timepoints during their development into iTreg or iTeff lines from naïve T cells as a starting population. This analysis defined three branching levels and showed that iTeff lines strongly diverge (i.e. reach branches 2 and 3) from the starting naïve population (branch 1) already by day 5 of culture, whereas iTregs reached branching levels 2 and 3 only after 12 days of culture (**Figures 2A, B**), thus taking much longer to branch off from the starting population. Also, iTreg lines at day 14 contained more cells remaining in the undifferentiated branch 1, compared to iTeff lines. Assessment of T cell phenotypes in the three branches (all timepoints together) demonstrated cells in branch 1 highly expressing CD45RA and CCR7 and overlapping in phenotype with the original naïve T cells (**Figure 2C**; **Supplementary Figure 5**). Cells in branches 2 and 3 lacked CD45RA and CD127 but whereas PD-1, GITR, and CXCR3 were expressed in branch 2, CD25 was expressed higher in branch 3 (**Supplementary Figure 5**). Furthermore, while iTreg and iTeff cells were present in all branches, their phenotypes within a branch showed differences (**Figure 2C**; **Supplementary Figure 6**), iTregs expressed higher CD27 and higher TIGIT in branch 2 and higher CD38 and CXCR3 in both branch 2 and 3 compared to iTeff, whereas iTeff cells showed a higher expression of HLA-DR and CD103 in branch 2 and 3 (**Figure 2C**).

### Diverging CD27 expression on iTregs versus iTeffs during their induction trajectory

**Figure 3:**
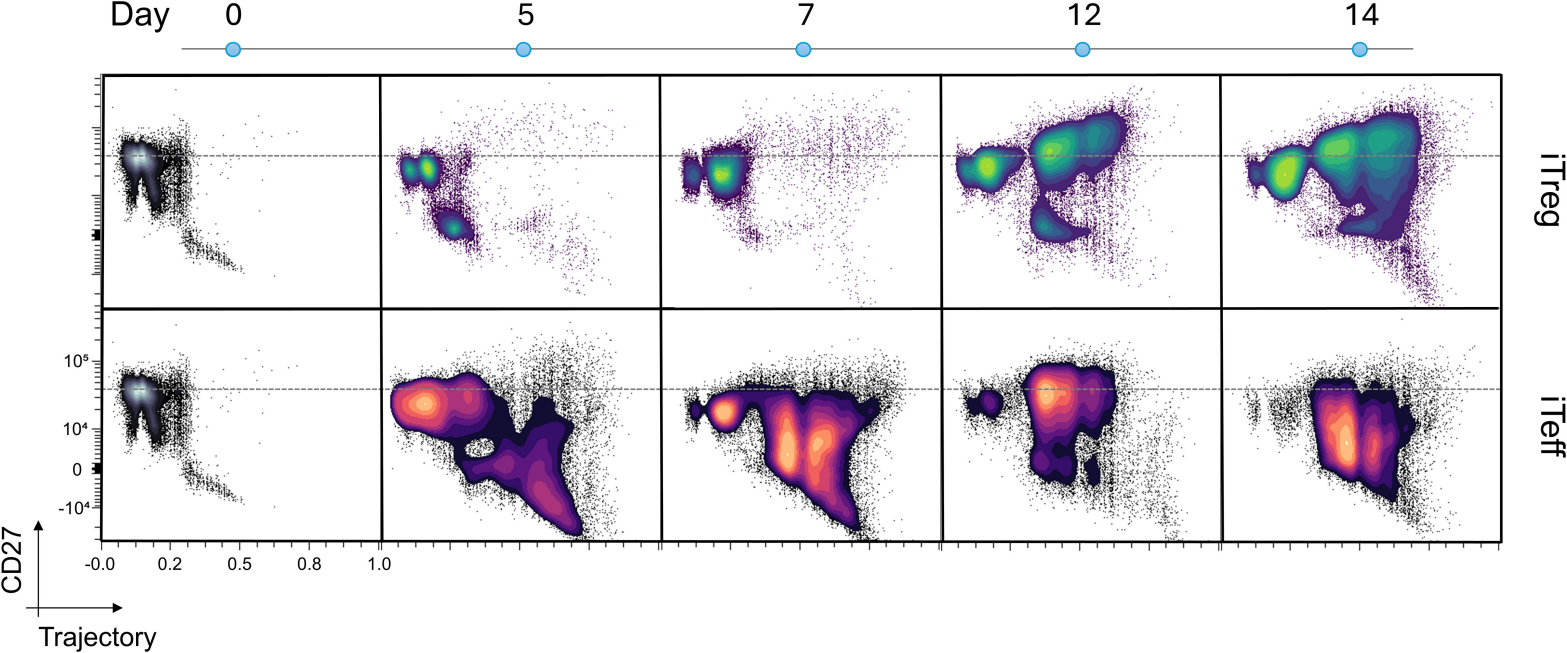
CD27 expression increases in iTregs cells with differentiation trajectory while CD27 expression decreases in iTeff cells. Trajectory analysis and CD27 expression in iTreg and iTeff cultures on different timepoints during the induction. Plots are shown as contour plots and dotted gray lines shows the CD27 expression on the spot where the abundance of naïve CD4 T cells is the highest on day 0. Representative data of one donor is shown.

Given the significant difference in CD27 expression between iTreg and iTeff when culturing was completed (**Figure 1**), we further defined the differentiation trajectories by Wishbone in relation to CD27 expression. Divergent CD27 expression and different trajectory kinetics were revealed between iTregs and iTeffs during their development (**Figure 3**), which is in line with the delayed start of branching in iTreg cultures (**Figure 2C**). The iTreg cultures reached their trajectory endpoint much later than iTeff lines (day 12 versus 5, respectively). This delayed differentiation trajectory for iTregs was confirmed by independent iTreg cultures generated from two different individuals (**Supplementary Figure 7**).

CD27 and CD45RA expression remained unchanged until day 12 in iTreg cultures, whereas iTeff cultures started moving along the trajectory already by day 5, reducing CD27 and CD45RA and upregulating CD25 (**Figure 3**; **Supplementary Figure 8**). The distinction between iTreg and iTeff lines became further pronounced on day 7, at which time the lines get restimulated, which is required for iTregs to obtain full suppressive characteristics (27). Indeed, five days after restimulation (day 12 after initiating the cultures) the iTreg cultures but not the iTeff cultures advanced in their trajectory with a steady increase in CD27 expression by memory-like iTregs, and this continued until the end of culture (day 14). Moreover, the increase in CD27 on memory-like iTregs was accompanied with TIGIT by the end of culture (**Figure 1**; **Supplementary Figure 8**). CD69, CD38, and GITR were expressed higher in iTregs approaching the trajectory end than iTeff cultures, whereas iTeff cultures express higher levels of HLA-DR than iTregs further in the trajectory (**Supplementary Figure 9**). The other markers tested did not show a divergent expression between iTregs and iTeff during differentiation.

**Figure 4:**
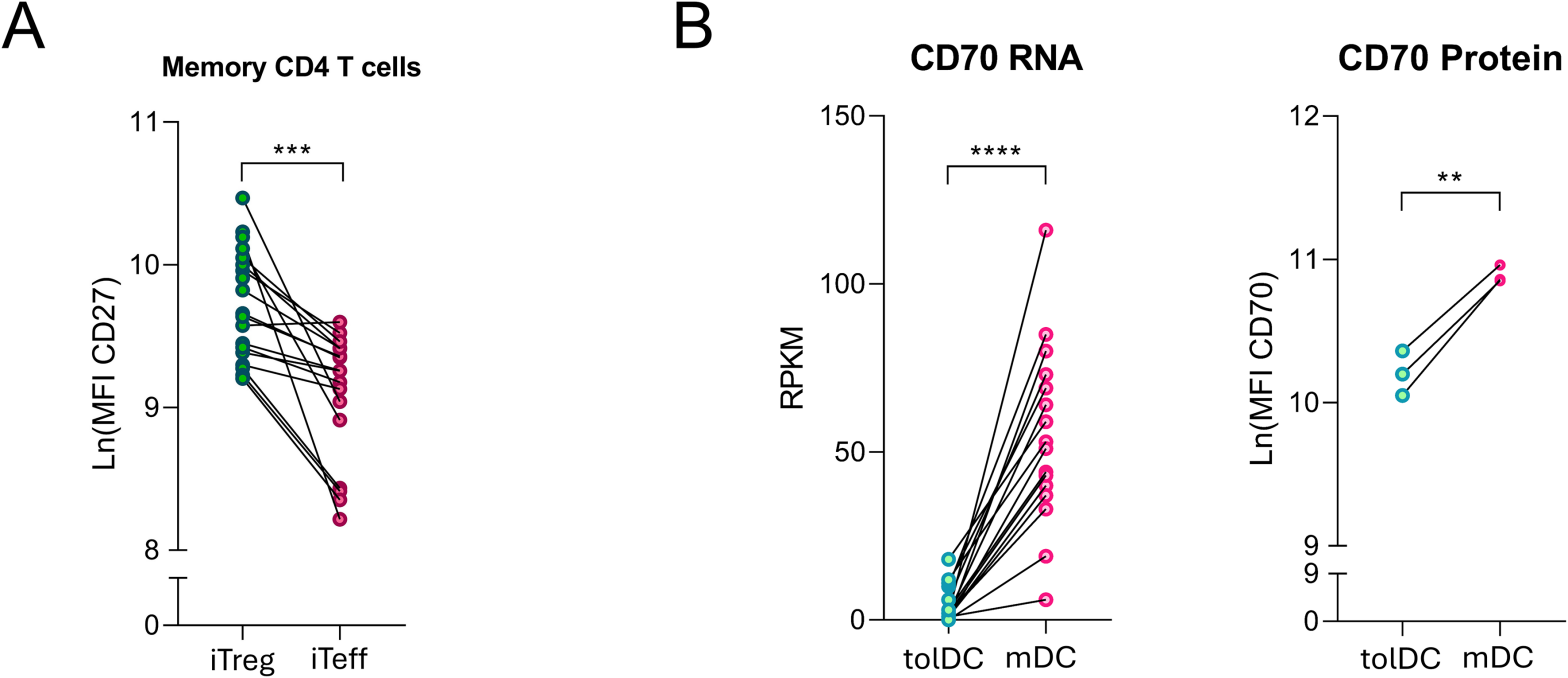
Memory-like iTregs express more CD27 as compared to iTeff cells on day 14 of culture. **(A)** Log-transformed mean fluorescence intensity of CD27 in memory T cells (CD45RAneg) from iTreg and iTeff cultures on day 14 of culture, from 17 donors cultured independently. **(B)** CD70 RNA (16 donors) and protein expression (3 donors) by dendritic cells (tolDCs and mDCs) generated in independent experiments. Paired Student’s t-test were performed to test for statistical difference between iTregs and iTeff or tolDC and mDC. Asterisks indicate **p<0.0021, ***p<0.0002 and ****p<0.0001.

Memory-like iTreg cultures have a significantly higher CD27 expression, a consistent phenotypic endpoint compared to memory-like iTeffs cultures from the same donor (p<0.0002, **Figure 4A**). In an attempt to explain the difference and divergence in CD27 expression trajectories between iTreg and iTeff cultures, we investigated the expression of the CD27 ligand, CD70, on DCs. Expression of CD27 on T cells is affected by its interaction with CD70 on antigen-presenting cells, which can lead to cleavage of the extracellular domain of CD27 which is internalized together with CD70 into the antigen-presenting cell (28). Indeed, CD70 expression on tolerogenic DCs was significantly lower than that on inflammatory DCs (**Figure 4B**), providing a potential explanation for the observed difference in CD27 expression between iTreg and iTeff lines.

### CD27+TIGIT+ iTregs correlate with suppressive capacity *in vitro* and ex vivo

**Figure 5:**
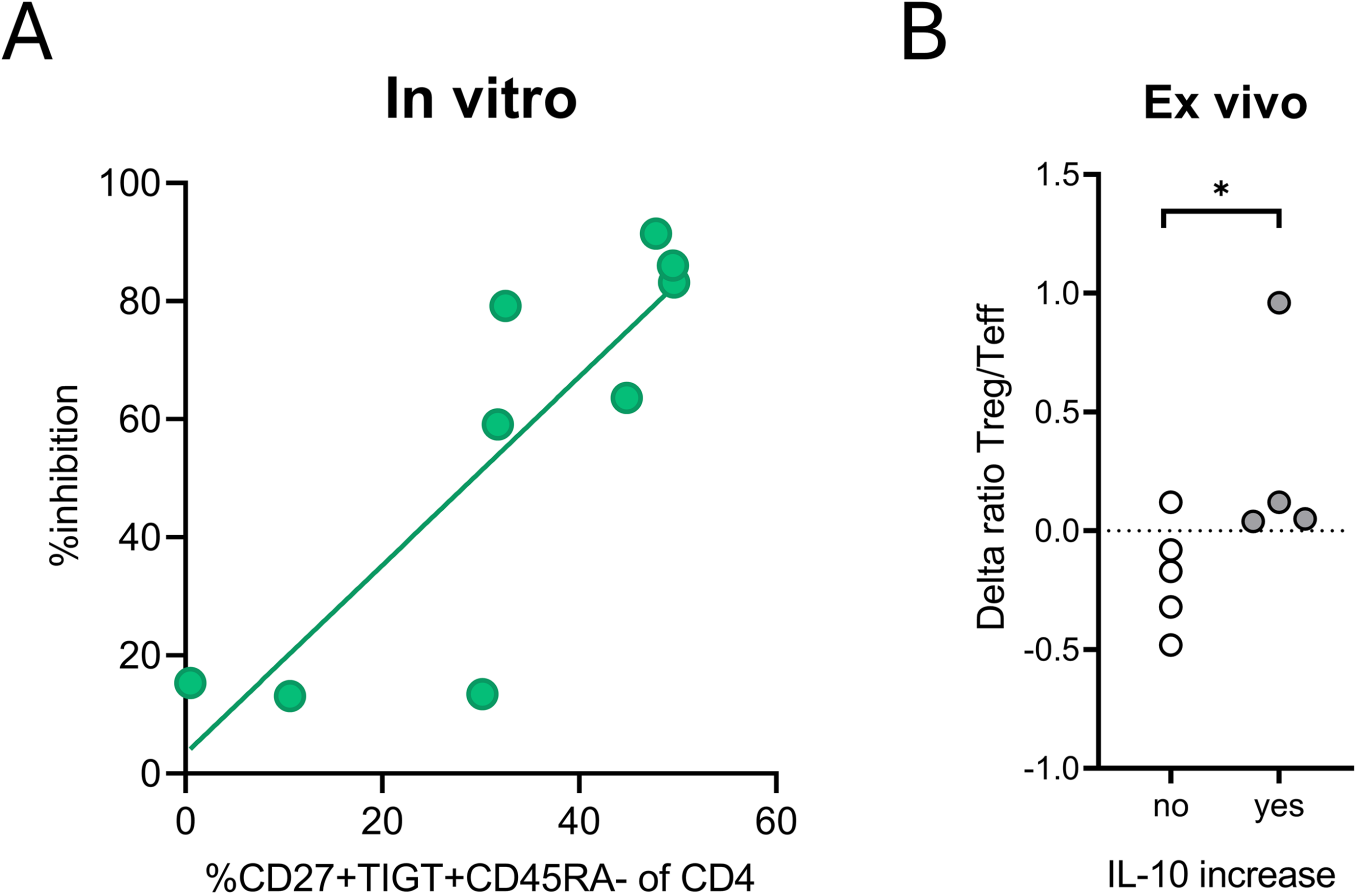
Abundance of CD27+TIGIT+ iTregs correlate with suppressive capacity *in vitro* and ex vivo. **(A)** Correlation between the abundance of CD27+TIGIT+ memory T cells within iTreg lines and their suppressive capacity. The suppressive capacity of each iTreg line was tested in a suppression assay, where autologous naïve CD4 T cells labeled with CFSE (responder cells) were stimulated with mDCs in de presence of iTregs or irradiated autologous naïve CD4 T cells as crowding control. Autologous naïve CFSE-labeled CD4 T cells without stimulation were taken along as no division control. Percent inhibition was calculated by taking the results of no division control as 100% inhibition and the proliferation of the mDC-stimulated responder cells in the presence of crowding control as no inhibition. Data is shown from nine distinct donors all performed in independent suppression assays. Correlation between abundance of CD27+TIGIT+ iTregs and their suppressive capacity *in vitro* was analyzed with linear regression analysis (R^2^ = 0.72, p=0.004). **(B)** Positive change in CD27 and TIGIT-expressing memory-like CD4 T cells is found in individuals showing immunological efficacy to reduce islet autoimmunity in a trial injecting tolDCs in T1D patients. Change in ratio of CD27+TIGIT+ CD4 T cells (similar to Tregs generated *in vitro*) and CD27lowCD45RAneg T cells (similar to Teff cells generated *in vitro*) after tolDC injection. Patients are divided into two groups based on their PBMC response to the vaccine peptide determined by ELISPOT assay: increased number of IL-10-producing cells (gray) and decreased or unchanged number of IL-10-producing PBMC (white). Unpaired student T test showed a significant difference (*p=0.037) in changes in ratio Treg/Teff in vivo between the two groups.

Finally, we investigated functional correlates of the CD27+TIGIT+ iTreg subset that we identified and assessed whether these cells pertain immune regulatory qualities. There was no correlation between the frequency of FOXP3 expressing cells and the CD27+TIGIT+ subset (**Supplementary Figure 2C**). We compared the amount of CD27+TIGIT+ memory-like cells in iTreg lines with their suppressive capacity *in vitro* (**Supplementary Figure 10**). An increased capacity to inhibit proliferation of naïve T cells was observed with an increasing rate of CD27+TIGIT+ iTregs (R^2^ = 0.72; p=0.004; **Figure 5A**). To explore whether our findings *in vitro* may have an *in vivo* correlate and potential application, we revisited our phenotyping and functional studies in a clinical trial in which T1D patients were vaccinated with tolerogenic DCs pulsed with islet autoantigen (25, 26). Indeed, a T cell population expressing low CD45RA, high CD27 and TIGIT was detectable in peripheral blood samples from treated patients that showed a temporary change after tolDC injection in 4 out of 9 patients (**Supplementary Figure 11**). Building on the different CD27 expression kinetics noted during induction of iTregs and iTeffs *in vitro* and appreciating that tolDC induce iTregs and eliminate Teff cells, we calculated the ratio between memory CD27+TIGIT+ T cells (potentially containing tolDC-induced iTregs) and CD27low memory T cells (Teff) (**Supplementary Table 3**). This ratio increased in the blood in the same 4 patients who also demonstrated increased IL-10 production by PBMCs in response to the proinsulin vaccine peptide (p= 0.037; **Figure 5B**). This supports the premise that the CD27+TIGIT+ CD4 population in peripheral blood contain induced antigen-specific iTregs and associate with induction of autoantigen-specific immune regulation.

## Discussion

Inducing antigen-specific Tregs are currently explored as therapeutic strategy for intervention in autoimmune diseases. Yet, phenotypic changes that mark the induction of antigen-specific Tregs are poorly defined. In this study, we monitored the expression of CD27 within a panel of markers associated with immune activation and regulation during priming of autoantigen-specific iTregs with tolDCs. We demonstrate that iTreg cultures induced *in vitro* consist of memory-like iTreg marked by a high CD27 expression as well as T cells that retain a naïve phenotype and lack suppressive capacity (24), which is distinct from memory-like Teff cultures following their priming by inflammatory DC. This diverging CD27 expression between iTreg vs. iTeff occurs early during the differentiation trajectory; where memory-like iTregs branch off after the second stimulation with tolerogenic DC and increase CD27 expression, in contrast to memory-like Teff cell cultures that arise soon after their first priming by inflammatory DCs and gradually lose CD27 expression. Given that CD27 is an important molecule in immune regulation by nTregs (10, 11), our results suggest that high CD27 expression is a characteristic of fully differentiated functional Tregs, irrespective of their origin or target antigen.

We previously pointed to a need to combine phenotype with specific functional properties to study Tregs categorically (7). We here demonstrate that the abundance of CD27hi expressing cells in iTreg lines indeed correlates with their suppressive capacity *in vitro*. CD27 enables Tregs to block CD70-mediated co-stimulation and thus contributes to regulation by changing the accessibility of co-stimulatory molecule CD70 on dendritic cells to curtail T cell activation. Indeed, CD27 on Tregs impairs priming of Th1 cells by downregulating CD70 expression on DCs in mice (28). Moreover, selective knockout of CD27 in Tregs impaired tolerance and facilitated CD8 T cell priming by DCs fostering CD8 mediated tumor control (29). Furthermore, in our studies CD27+ Tregs co-express higher levels of TIGIT, GITR, CD69 and CD38, which all have been described to either support Treg suppressive function or contribute to Treg survival (30–34). Our observation that CD27 follows regulation and reduces with inflammation underscores the association on CD27 with cancer progression and impaired anti-tumor immunity.

Of the Treg-associated molecules included in our study, CD27+ TIGIT+ iTregs express PD1, LAG3 and CD103 during iTreg differentiation *in vitro*. When expressed on Tregs, CD103 marks cells with superior suppressive function compared to the CD103 negative counterparts (35, 36). After tolDC injection in T1D patients, the percentage of CD4+CD103+ T cells increased significantly in the circulation (25). This CD103+ population consisted of around 20% of CD27+TIGIT+ cells, pointing to partial overlap with the subset resembling our iTregs. While the panel used for phenotype analysis included many Treg-related molecules, it is not exhaustive. Intracellular FOXP3 often used to identify Tregs, which is evident for mouse or thymus derived Tregs, but not for all induced Tregs in humans (37–43). Our tolDC-induced antigen-specific Tregs show variable expression of FOXP3 protein and partial demethylation of the FOXP3 promotor and resemble the Tr1 cells in this respect. Since all stimulated human T cells also express FOXP3 protein regardless of their regulatory capacity (7), which we also observed in this study, this disqualified FOXP3 for discrimination between *in vitro* activated iTeff and iTreg. Furthermore, the permeabilization protocol required for an intracellular FOXP3 staining could not be combined with our current multidimensional flow cytometry. Surface molecules, such as ectoenzymes CD39 and CD73 (44, 45), remain to be examined in the trajectory of *in vitro* Teff and Treg differentiation. In our tolDC treated patients, however, CD27+TIGIT+ CD4 T cells all express CD39 (25).

Our findings of the divergent CD27 expression between Teff and Treg cells when stimulated with tolerogenic versus inflammatory DCs align with the literature showing that nTreg maturation in the thymus is also marked by the acquisition of CD27, where controlled signaling through CD27 upon interaction with CD70 on thymic epithelial cells and DCs enhances positive selection of Treg cells (46, 47). The low expression of CD70 by tolerogenic DC could similarly provide limited interaction with CD27 on naïve T cells and thus support their development into Tregs. The importance of CD27 and CD70 interaction for Treg differentiation is reinforced by the study in mice showing that genetic ablation of CD27 or CD70 in mice reduced Treg numbers in the thymus and peripheral lymphoid organs while leaving Teff cell numbers unaffected (47). The interaction of CD27 with CD70 can also lead to CD27 cleavage from the T cell membrane, possibly explaining our observation that naïve T cells rapidly lost CD27 expression upon interaction with inflammatory DC that highly express CD70, whereas the low expression of CD70 on tolerogenic DC allowed sustained CD27 expression by iTregs.

Tumors employ the CD27-CD70 axis to mediate immune escape through its effects on Tregs. CD27 expressing tumor infiltrating lymphocytes (TILs) surrounded CD70-expressing tumor cells and secrete high amounts of IL-10 and TGF-beta, creating an immunosuppressive tumor microenvironment (48, 49). The relation between CD27 expression and immune regulation has been investigated in several diseases besides cancer. CD27 expression correlated with natural or therapy-induced tolerance; pollen-specific effector CD4 T cells lacked CD27 expression in allergic patients but expressed CD27 in tolerant individuals. Low frequencies of CD27 expressing T cells in patients with a pollen allergy increased gradually during tolerance inducing immunotherapy (50). In the context of autoimmune diseases, expression of CD27 on CD4+CD25+ T cells in peripheral blood and synovial fluid of idiopathic juvenile arthritis patients correlated with suppressive activity (11, 51). Furthermore, type 1 diabetes patients have lower percentages of CD45RO+CD27+ T cells in peripheral blood than non-diabetic controls (52). In our clinical trial injecting tolDCs in type 1 diabetes patients, increasing frequencies of CD27+TIGIT+ T cells relative to effector T cells correlated with immunological efficacy measured by the increased production of IL-10 in response to islet autoantigens. Therefore, monitoring of CD27+ Tregs could be useful for cancer prognosis as well as provide an immune correlate of therapeutic efficacy in clinical trials aiming at antigen- or tissue-specific immune regulation.

In summary, our results show an increased CD27 expression during iTreg induction that diverges from Teff cells and correlates with good suppressive function. The level of CD27 expression in combination with TIGIT may mark functional Tregs irrespective of the suppressive mechanism they utilize. Furthermore, *in vivo* detection of CD27 and TIGIT expressing memory Treg cells might be helpful as an immune correlate for tolerance induction in the clinic.

## Data Availability

The raw data supporting the conclusions of this article will be made available by the authors, without undue reservation.
