# Supplementary material for: Divergent CD27 expression marks the Treg induction trajectory
Source: Front Immunol. 2026 Mar 9;17:1756275. doi: 10.3389/fimmu.2026.1756275 (PMC13006287; doi:10.3389/fimmu.2026.1756275)
Supplement: Supplementary file 1 [file DataSheet1.docx]

Supplementary Material

**Supplementary Table 1**: Antibody surface panel used for phenotype analysis with spectral flow cytometry

| **Marker** | **Fluorochrome** | **Clone** | **Company** |
| --- | --- | --- | --- |
| CD27 | APC | M-T271 | BD Bioscience |
| ICOS | PerCP Cy5.5 | C398 | Biolegend |
| GITR | FITC | 110416 | R&D Systems |
| CD7 | PE-Cy5 | CD7-6B7 | Biolegend |
| CD69 | BV510 | FN50 | BD Bioscience |
| Lag-3 | BUV661 | 3DS223H | Invitrogen |
| LAP | eFluor450 | FNLAP | Invitrogen |
| CD103 | BUV563 | BER-ACT8 | BD Bioscience |
| CD56 | BUV737 | NCAM16.2 | BD Bioscience |
| PD-1 | BV421 | EH12.2H7 | Biolegend |
| CXCR3 | BV650 | G025H7 | Biolegend |
| CCR6 | BV711 | G034E3 | Biolegend |
| CCR7 | BV785 | G043H7 | Biolegend |
| CD127 | APC-R700 | HIL-7R-M21 | BD Bioscience |
| CD107a | PerCP-eF710 | eBioH4A3 | Invitrogen |
| KLRG1 | PE-Fire810 | SA231A2 | Biolegend |
| CD19 | BUV737 | H1B19 | BD Bioscience |
| CXCR5 | BV750 | J252D4 | Biolegend |
| CTLA4 | PE Dazzle 594 | L3D10 | Biolegend |
| CD49b | BV480 | AK-7 | BD Bioscience |
| CD226 | BV605 | 11A8 | Biolegend |
| HLA-DR | BV570 | L243 | Biolegend |
| CD38 | APC-eFluor780 | HIT2 | Invitrogen |
| CCR4 | PE | 1G1 | BD Bioscience |
| CD25 | PE Cy7 | M-A251 | BD Biosciences |
| CD25 | PE Cy7 | 2A3 | BD Biosciences |
| TIGIT | AlexaFluor647 | A15153G | Biolegend |
| CD45RA | BUV395 | HI100 | BD Bioscience |
| CD3 | BUV496 | okt-03 | BD Bioscience |
| CD8 | Qdot 800 | 3B5 | Invitrogen |
| CD6 | BUV805 | M-T605 | BD Bioscience |
| CD14 | BUV737 | M5E2 | BD Bioscience |
| CD4 | CF568 | SK3 | Cytekbio |
| LIVE/DEAD | Blue | - | Invitrogen |


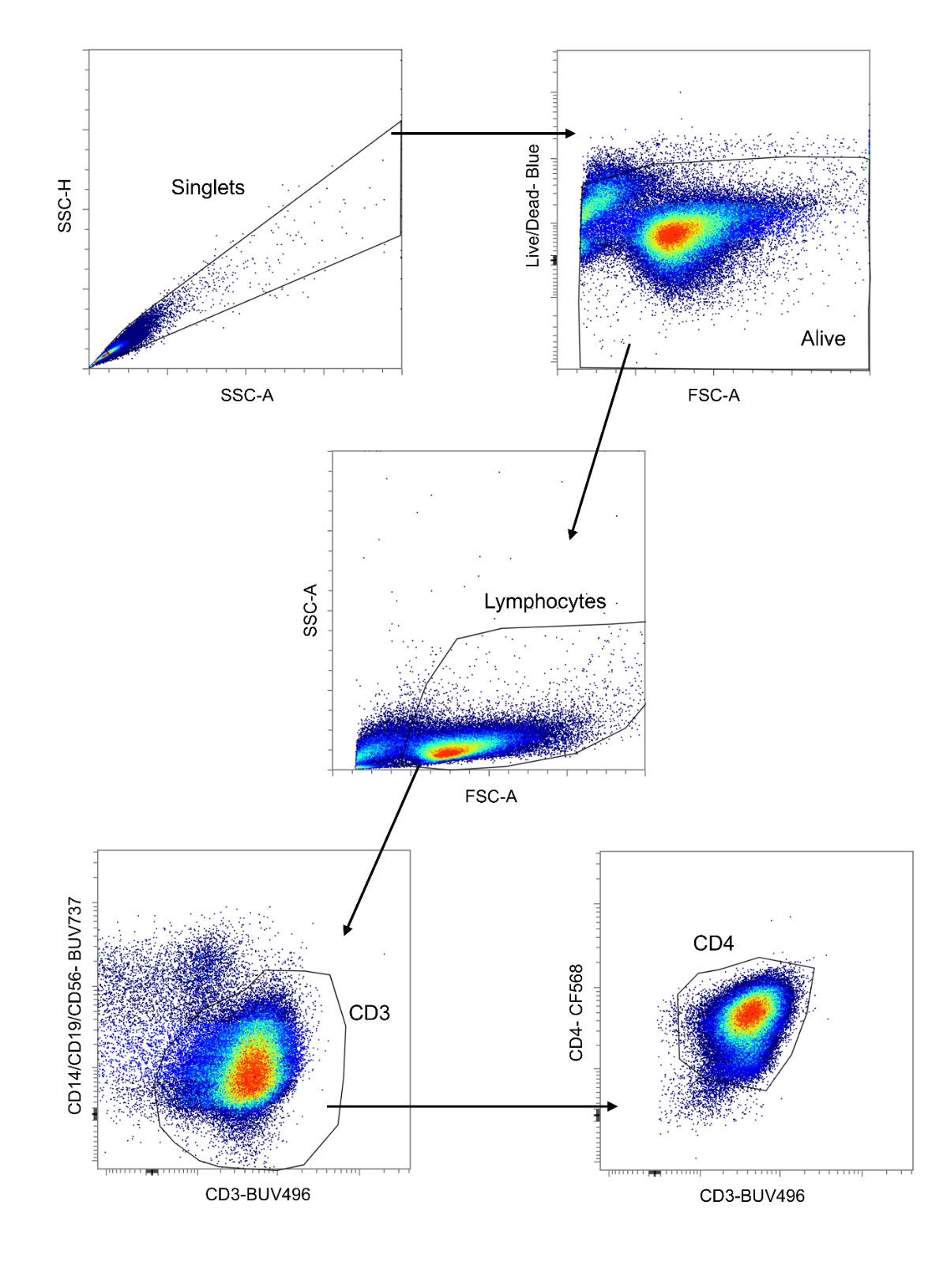


**Supplementary Figure 1**: Gating strategy before clustering (optnse) analysis.

**Supplementary Table 2**: Antibody intracellular panel used for phenotype analysis with spectral flow cytometry

| **Marker** | **Fluorochrome** | **Clone** | **Company** |
| --- | --- | --- | --- |
| CD45RA | BV785 | HI100 | BioLegend |
| CD15s | BV510 | CSLEX1 | BD Bioscience |
| CD3 | BV605 | OKT3 | BioLegend |
| CD4 | BV650 | SK3 | BD Bioscience |
| CD8 | PE-Dazzle594 | SK1 | BioLegend |
| CD25 | PE | M-A251 | BD Bioscience |
| FOXP3 | AF647 | 259D | Beckman Coulter |
| Helios | Pacific Blue | 22F6 | BioLegend |
| Ki67 | FITC | B56 | BD Bioscience |
| CD69 | PE-Cy7 | FN50 | BioLegend |


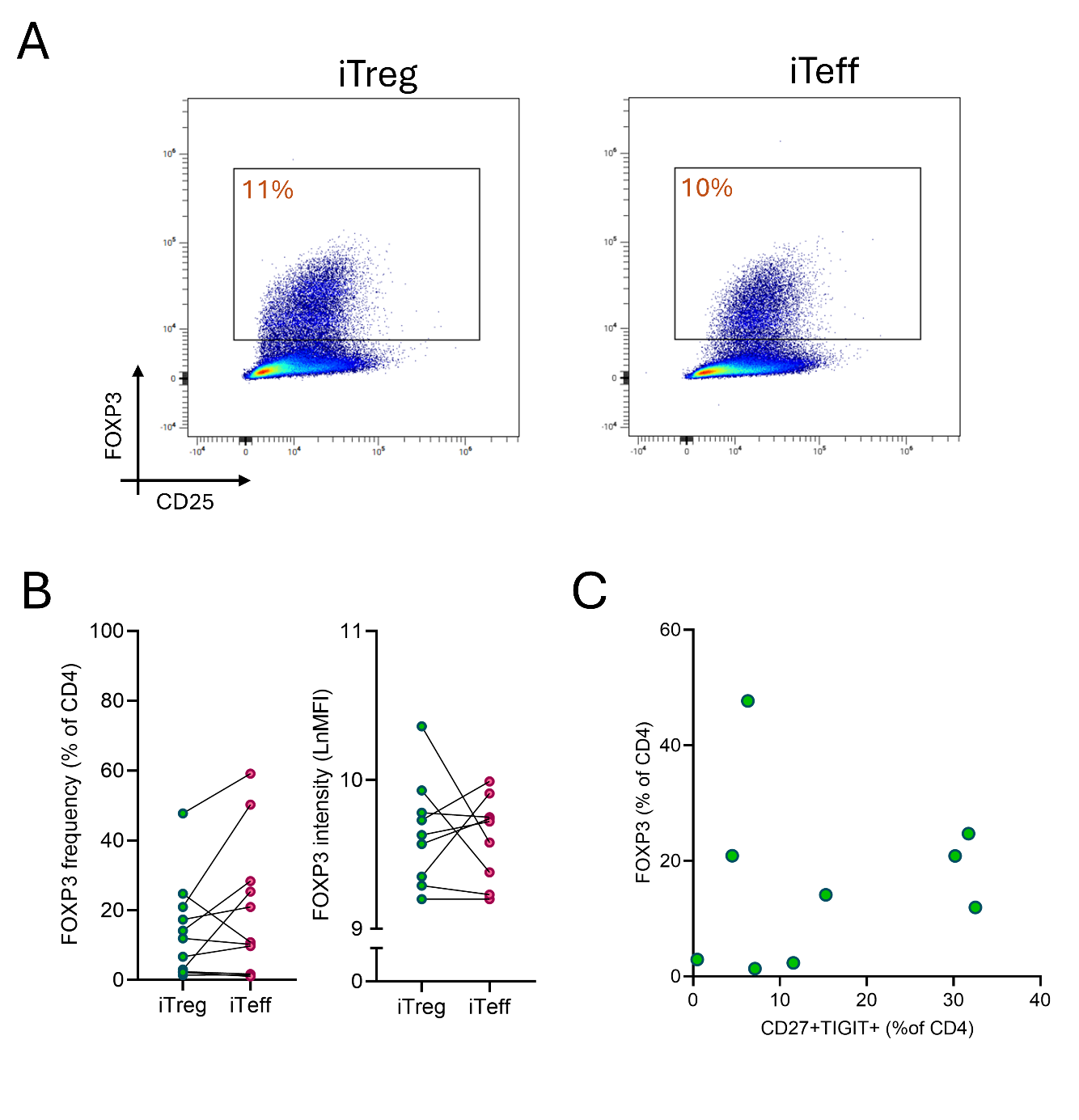


**Supplementary Figure 2:** FOXP3 expression within iTreg or iTeff lines and its correlation with CD27 expression. A) Representative intracellular FOXP3 staining from one donor, with frequency of FOXP3+ CD4 T cells indicated in top left corner per line. B) Frequency (left graph) and mean fluorescence intensity (right graph) of FOXP3 expression between paired iTreg or iTeff lines from nine independent donors on day 14 of culture. Paired Student’s t-test showed no statistical difference between iTreg and iTeff lines in frequencies (p=0.13) or expression level (p=0.79) of FOXP3. C) Correlation between the abundance of CD27+TIGIT+ T cells (surface staining) and the abundance of FOXP3+ T cells (intracellular staining) for iTreg lines from nine independent donors on day 14.


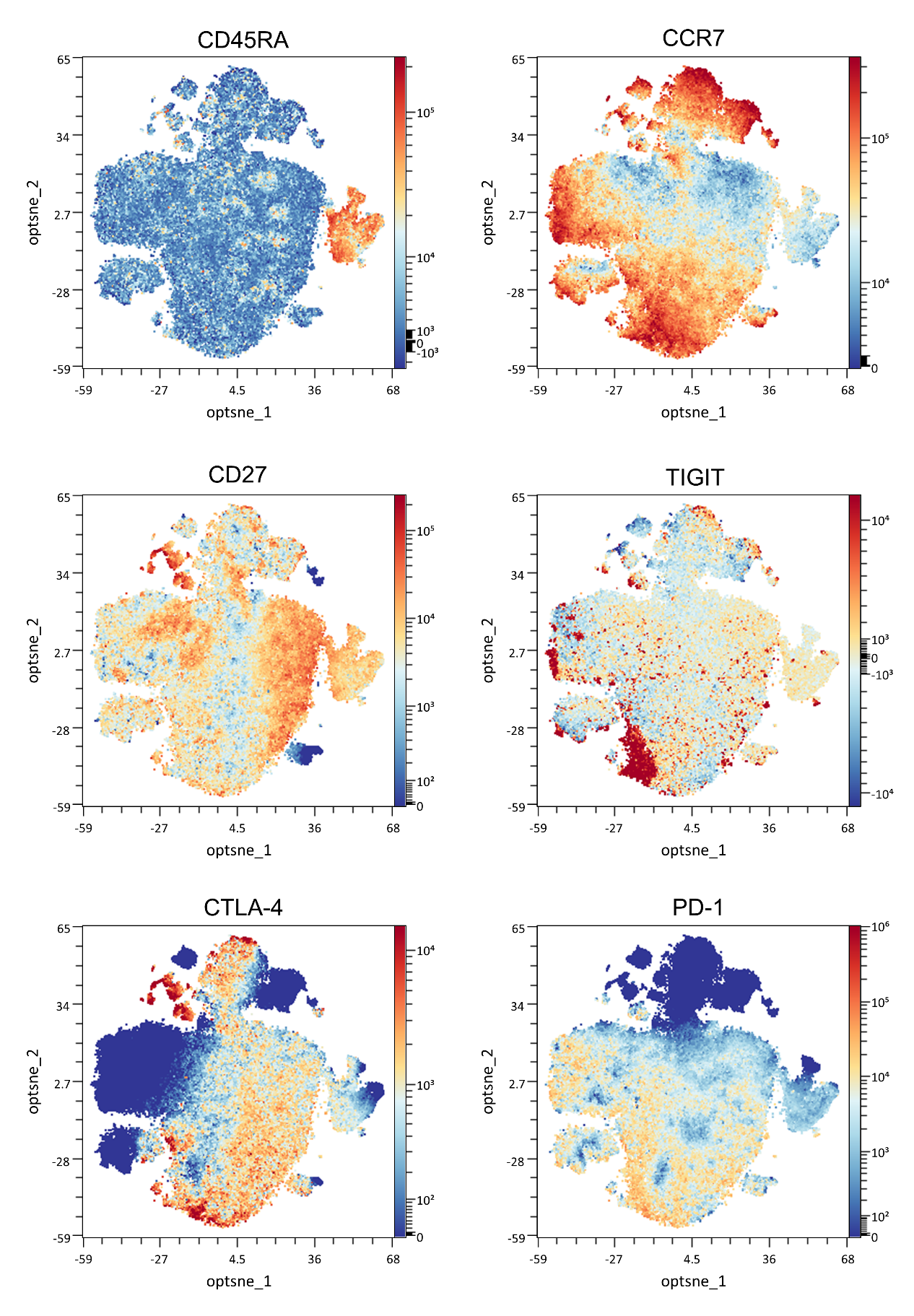


**Supplementary Figure 3**: Marker expression within the optsne cluster analysis. Colors depict level of expression ranging from low (blue) to high (red).


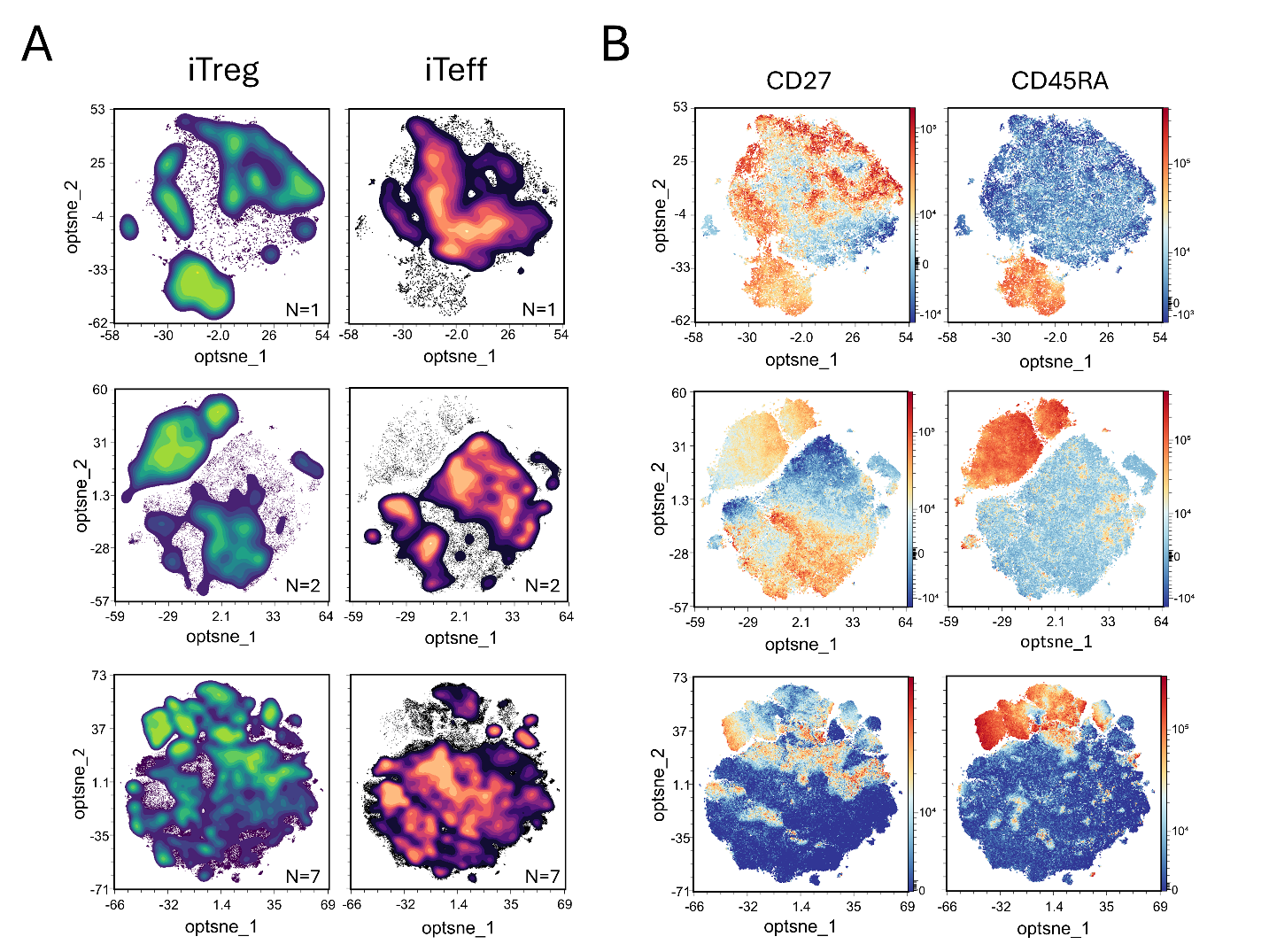


**Supplementary Figure 4**: Clustering analysis (optsne) of iTreg and iTeff cells phenotype in three independently generated sets of data (N indicates number of donors per experiment). TolDC induced T cell lines (iTreg) show higher CD27 expression, especially in memory-like cells as compared to mDC induced T cell lines (iTeff). A) iTreg and iTeff plots are shown as contour plots. B) Show the CD27 and CD45RA expression within the clustering per experiment. Colors depict the level of expression ranging from low (blue) to high (red).


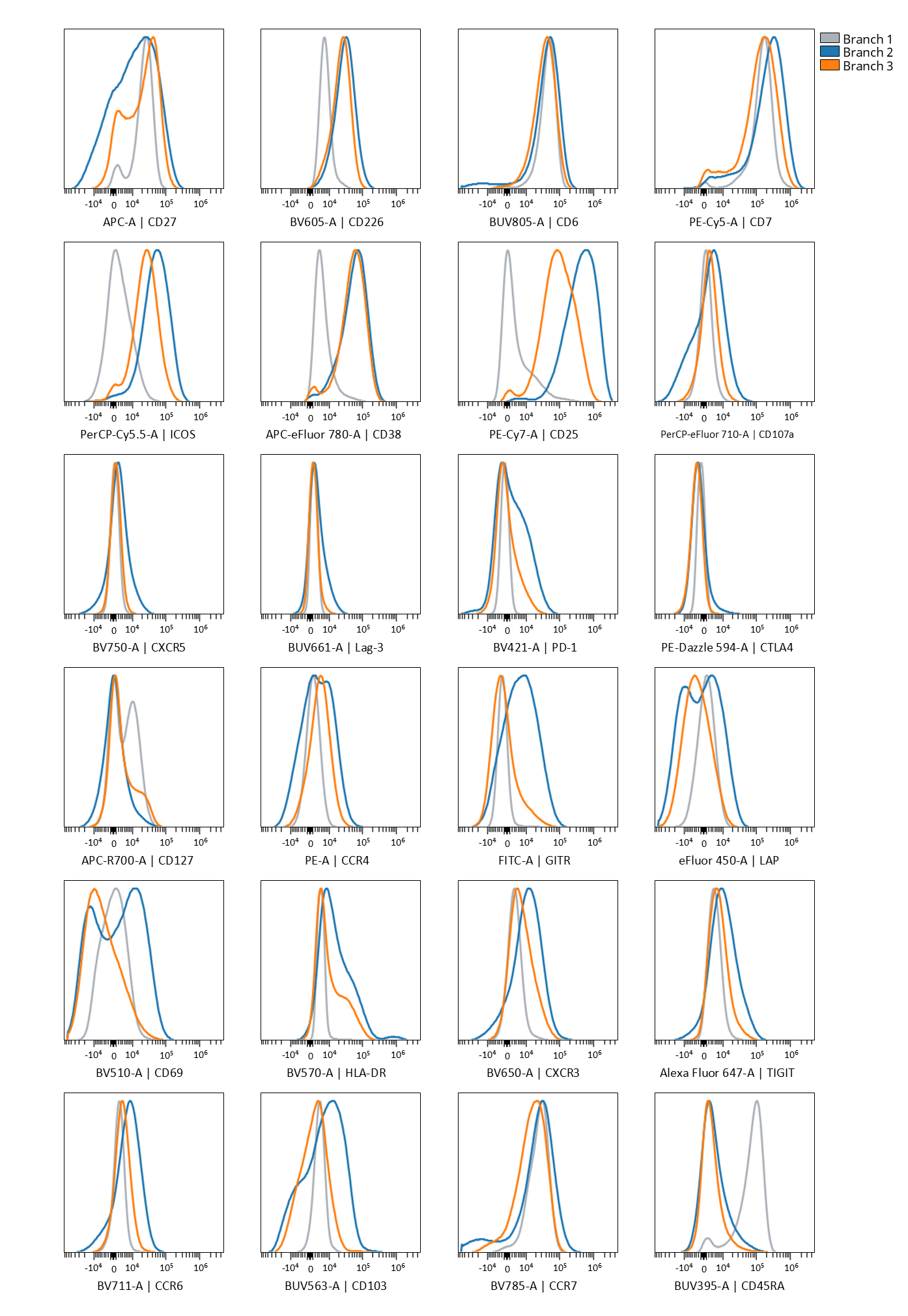


**Supplementary Figure 5**: Histograms showing the marker expression per branch (branch 1 (grey), branch 2 (blue) and branch 3 (orange)) as defined by Wishbone branching. Each branch consists of both iTregs and iTeff cells from all timepoints.


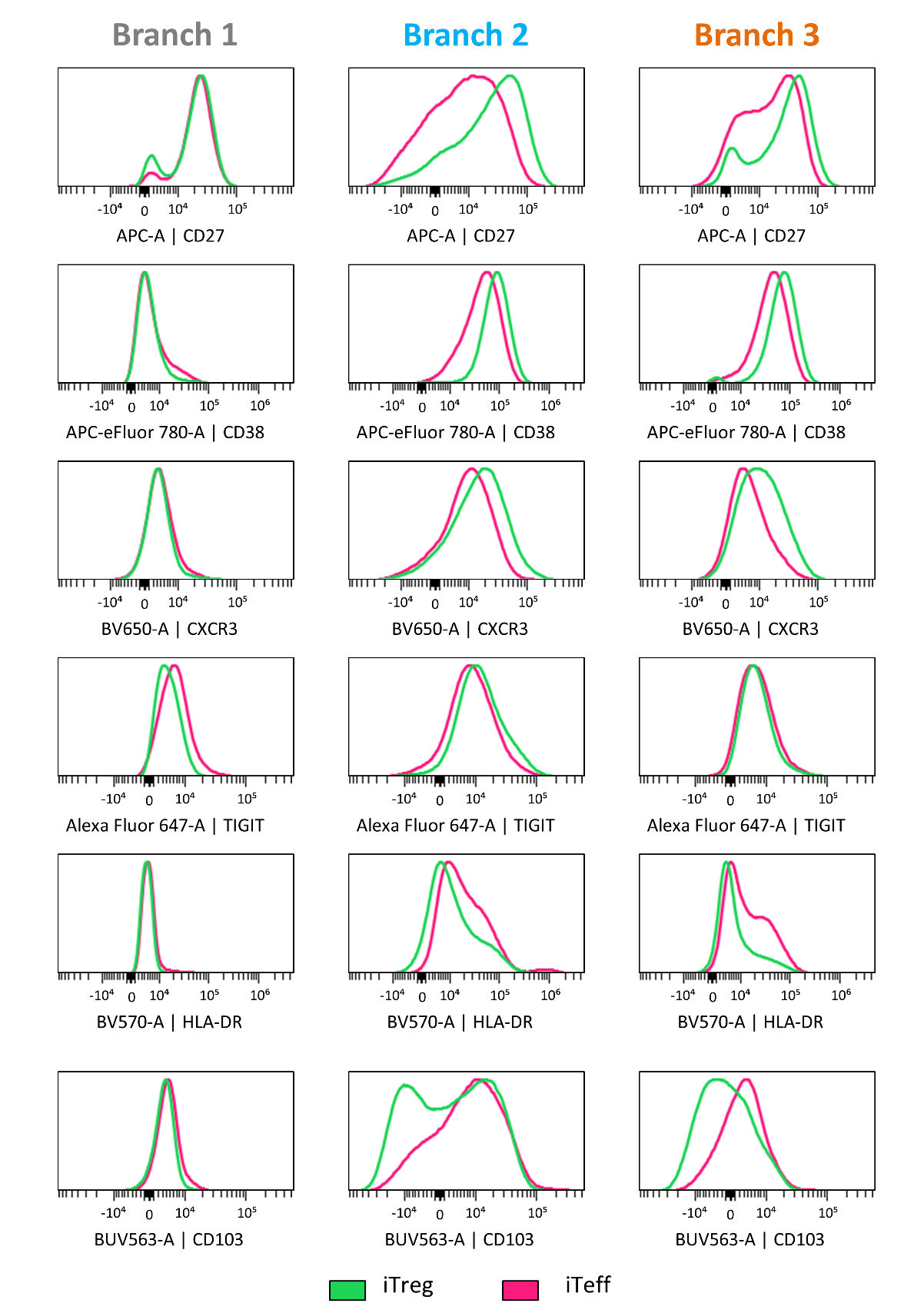


**Supplementary Figure 6:** Histograms showing the marker expression per branch separated in iTregs (green) and iTeff (pink) lines from all timepoints.


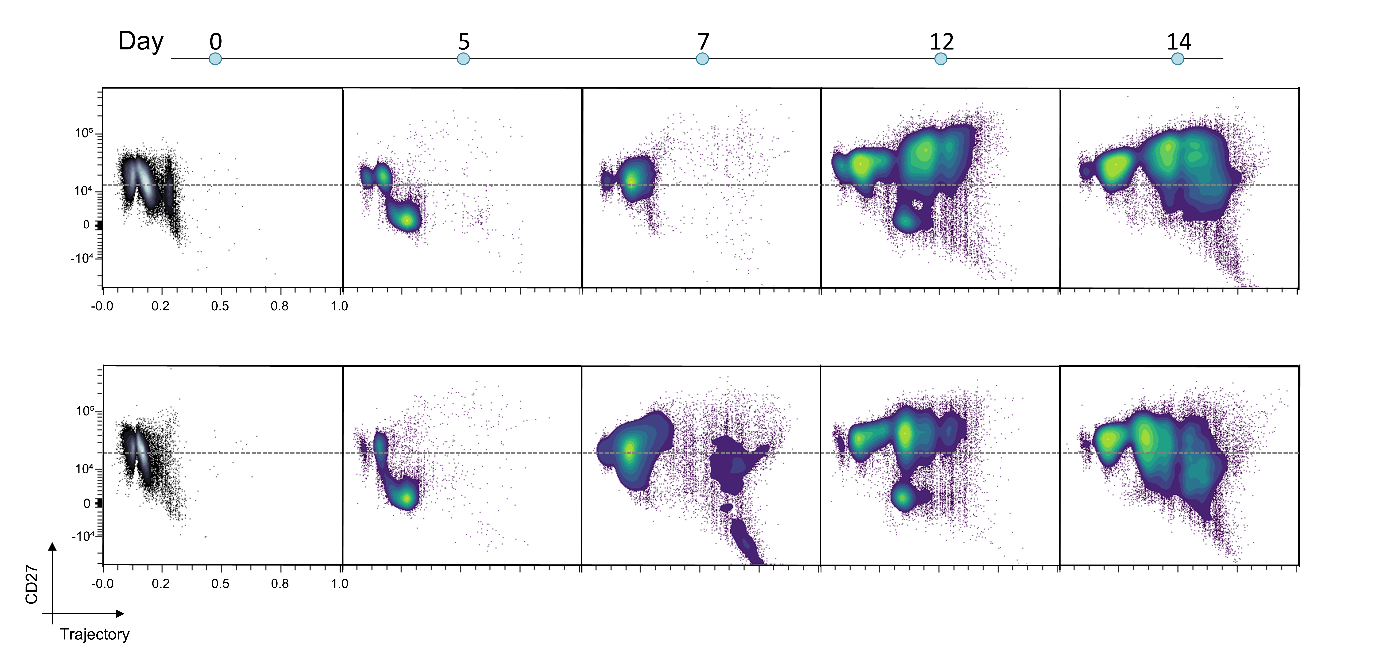


**Supplementary Figure 7**: iTreg lines from two additional independent donors showing increasing CD27 expression in iTreg cells with differentiation. Plots are shown as contour plots and dotted grey lines shows the mean CD27 expression on naïve CD4 T cells on day 0.


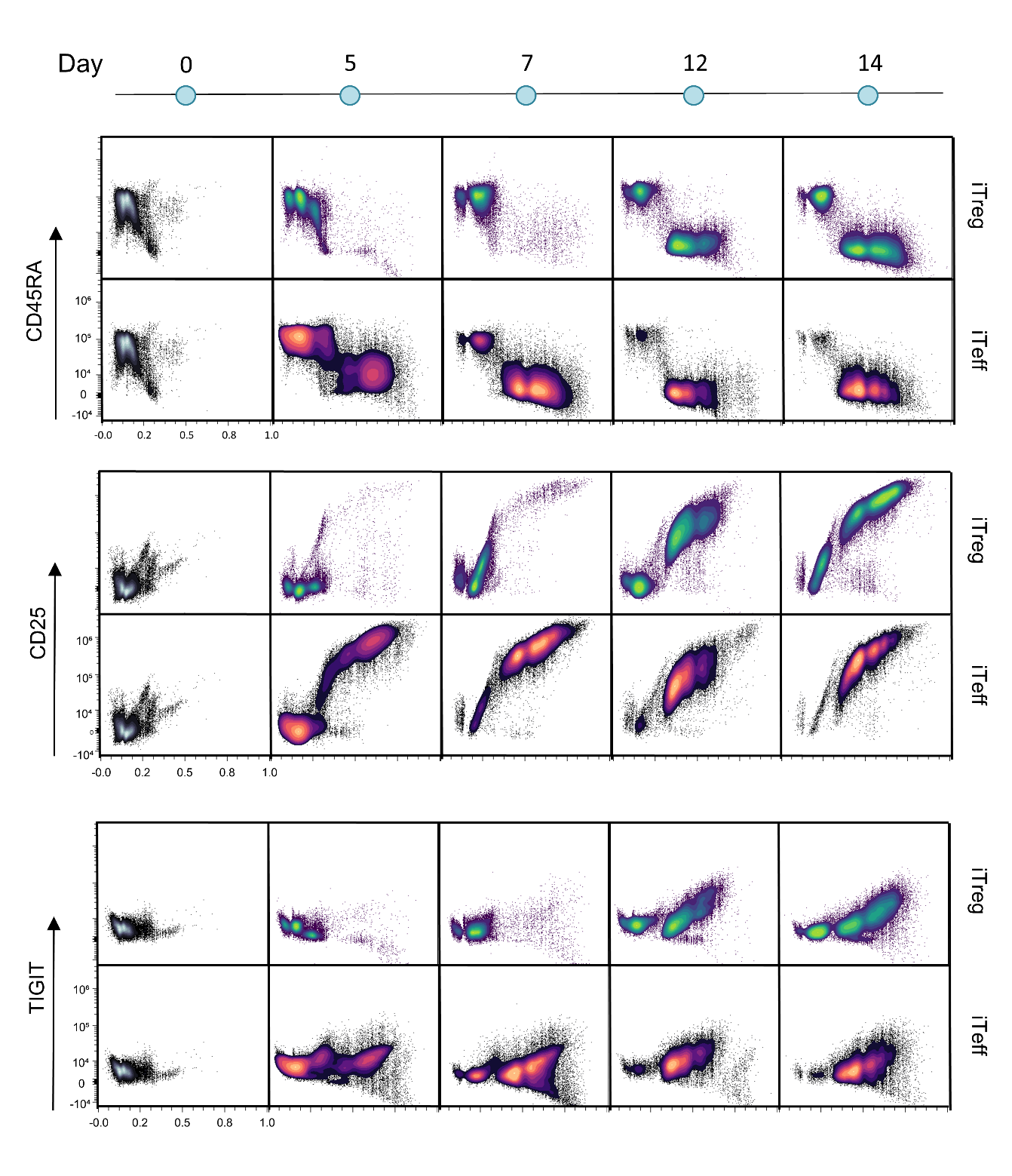


**Supplementary Figure 8**: Selected marker expression within the trajectory analysis at different timepoints during the induction of iTreg cells (green) or iTeff cells (orange). Data is shown from representative donor.


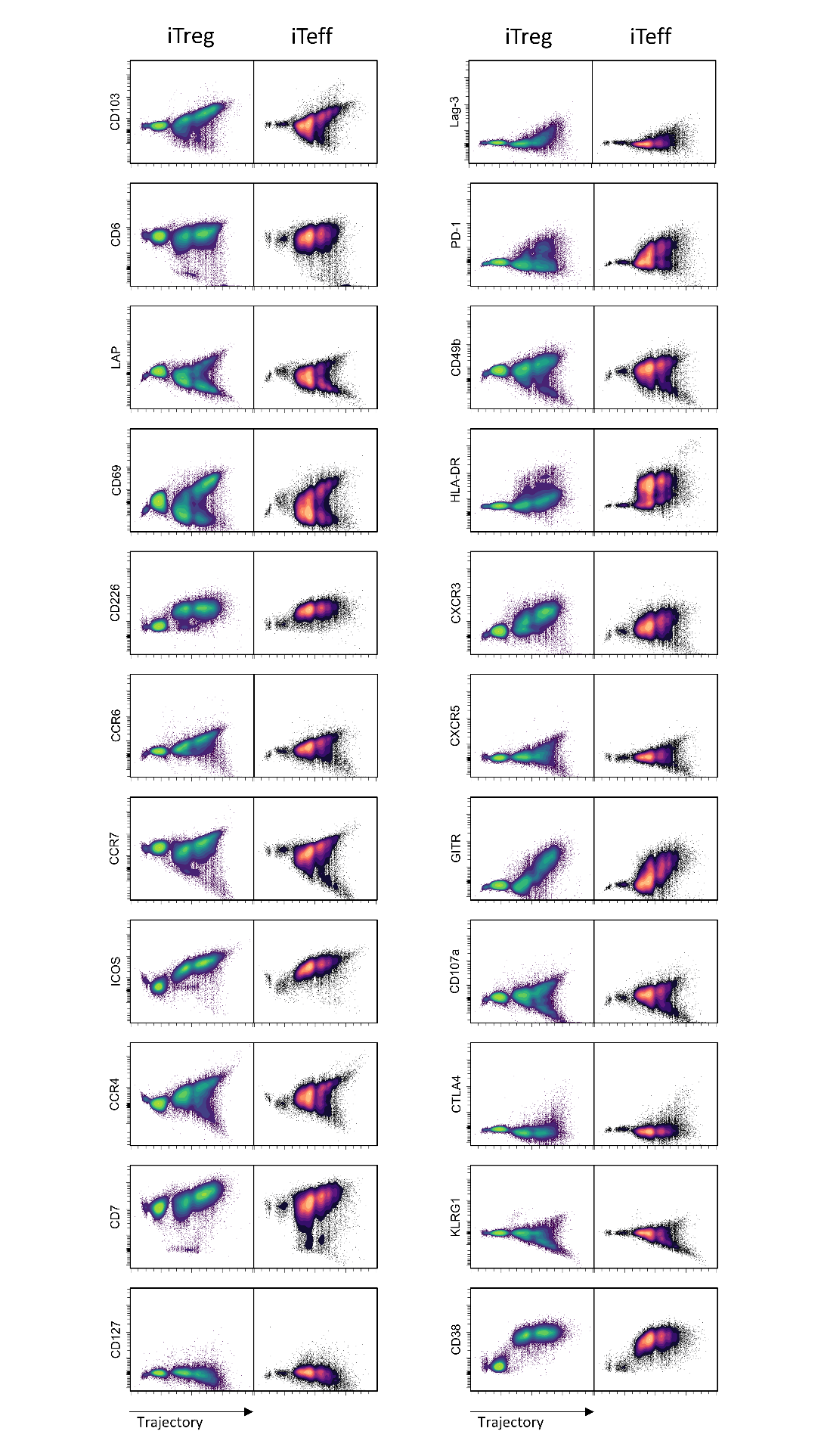


**Supplementary Figure 9**: Marker expression of all analyzed surface molecules (Supplementary Table 1) at day 14 within the calculated trajectory of iTreg cells (green) or iTeff cells (orange). Data is shown from representative donor.


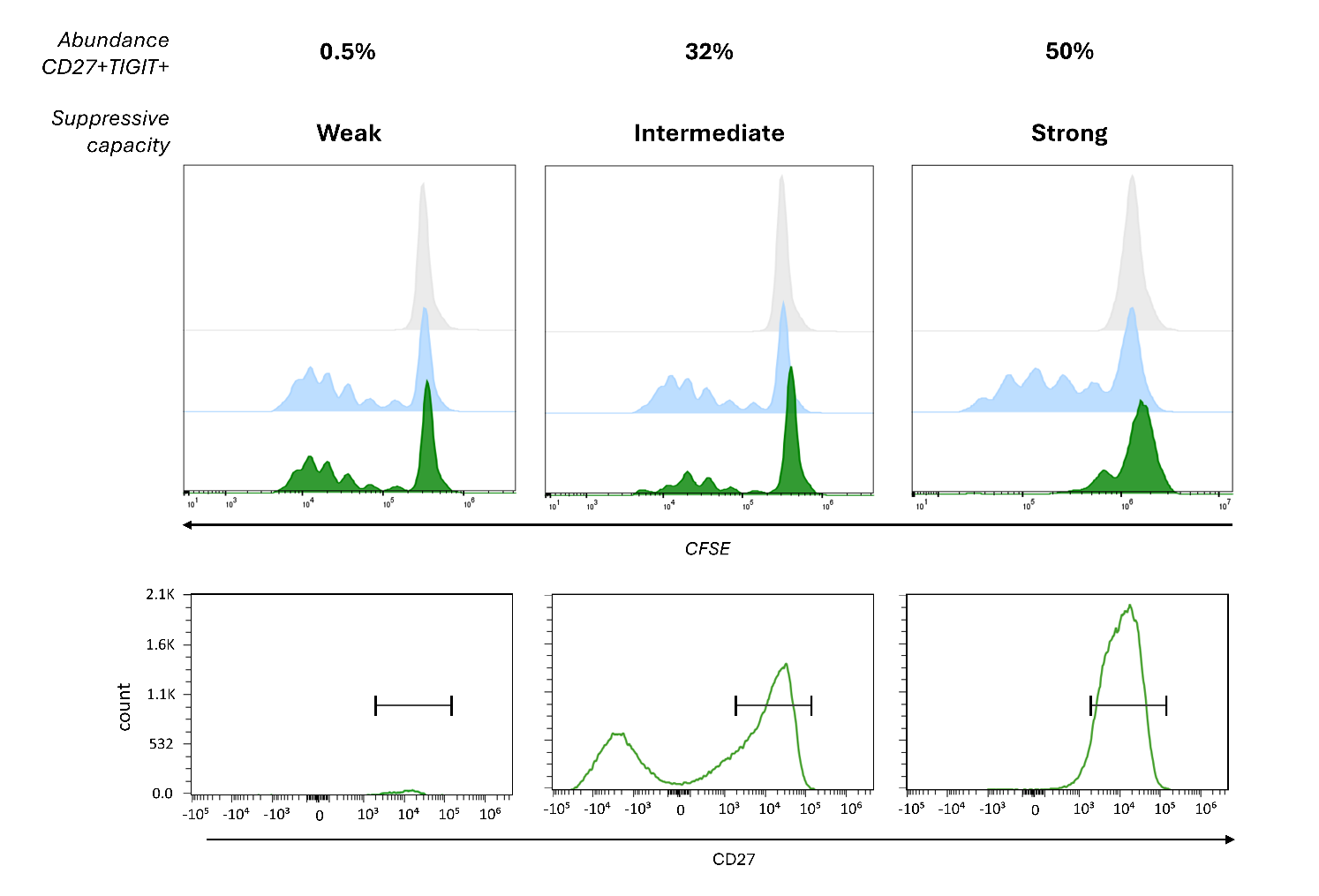


**Supplementary Figure 10:** Top panel shows representative FACS plots of the CFSE-labelled naïve responder T cell proliferation of iTreg lines (green filled histogram) that show weak, intermediate or strong suppressive capacity which corresponds to the low, intermediate and high abundance CD27+TIGIT+ memory T cells within the respective iTreg line. No stimulation control (no dendritic cells (DCs)) are shown as grey histrograms, stimulated responder cells by DCs are shown in blue histograms (maximal proliferation).The bottom panel shows CD27 expression histograms by memory T cells of the respective iTreg lines


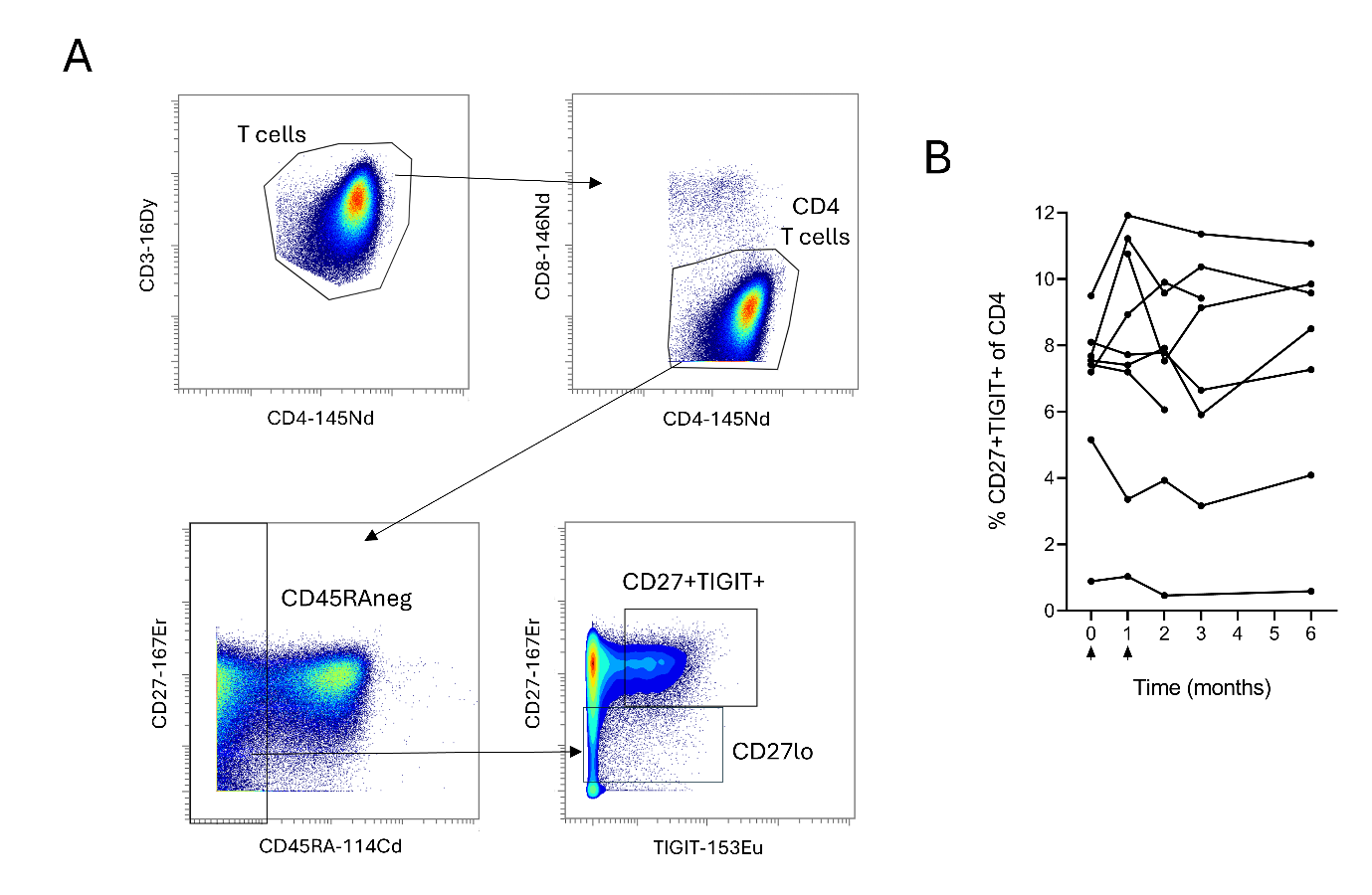


**Supplementary Figure 11**: In vivo detection of CD27+TIGIT+ CD45RAneg memory CD4 T cells in type 1 diabetes patients injected with tolDCs. A) Gating strategy of in vivo CD27+TIGIT+ CD45RAneg memory CD4 T cells. B) Abundance of CD27+TIGIT+ CD45RAneg within CD4 T cells before and after tolDC injection (arrows indicate timepoints of tolDC injection).

**Supplementary Table 3:** Absolute numbers(#) of CD27+TIGIT+ CD4 T cells (similar to Tregs generated in vitro) and CD27lowCD45RAneg T cells (similar to Teff cells generated in vitro) used to calculate a Treg/Teff ratio.

|  | Before | | | After | | |  |
| --- | --- | --- | --- | --- | --- | --- | --- |
| Patient Number | **#Treg** | **#Teff** | **Ratio** | **#Treg** | **#Teff** | **Ratio** | **Delta ratio** |
| 1-3 | 13605 | 26816 | *0.51* | 11450 | 59896 | *0.19* | *-0.32* |
| 2-3 | 12059 | 17591 | *0.69* | 16750 | 32513 | *0.52* | *-0.17* |
| 3-3 | 13383 | 22746 | *0.59* | 18060 | 35800 | *0.50* | *-0.08* |
| 1-2 | 9412 | 15474 | *0.61* | 27454 | 17518 | *1.57* | *0.96* |
| 1-1 | 1741 | 5198 | *0.33* | 1553 | 3393 | *0.46* | *0.12* |
| 3-2 | 11843 | 30625 | *0.39* | 18100 | 42657 | *0.42* | *0.04* |
| 2-1 | 15063 | 31176 | *0.48* | 21490 | 40311 | *0.53* | *0.05* |
| 3-1 | 24340 | 54794 | *0.44* | 26179 | 46429 | *0.56* | *0.12* |
| 2-2 | 13823 | 12166 | *1.14* | 8252 | 12519 | *0.66* | *-0.48* |
